# Immersive Virtual Reality in Alleviating Pain and Anxiety in Children During Immunization in Primary Care: A Pilot Randomized Controlled Trial

**DOI:** 10.3389/fped.2022.847257

**Published:** 2022-03-25

**Authors:** Zi Ying Chang, Gary Chun-Yun Kang, Eileen Yi Ling Koh, Rodney Jin Kai Fong, Jiasheng Tang, Chi Keong Goh, Ngiap Chuan Tan

**Affiliations:** ^1^SingHealth Polyclinics, Singapore, Singapore; ^2^SingHealth Duke-NUS Family Medicine Academic Clinical Program, Singapore, Singapore; ^3^AI Innovation Labs, Singapore, Singapore

**Keywords:** virtual reality, immunization, children, parent, nurse, pain, anxiety

## Abstract

**Background:**

Pediatric immunization is often associated with significant fear and anxiety among the children and their parents. Their distress may potentially affect their adherence to the childhood immunization schedule and the acceptance of other recommended vaccines by physicians.

**Objective:**

The study primarily aimed to assess the feasibility of using immersive virtual reality (VR) during immunization in children in primary care. The secondary aim was to determine the effectiveness of immersive VR in alleviating pain and anxiety among children, reduction of anxiety of their parents and attending nurses during immunization compared to usual care without VR.

**Methods:**

A pilot open-label randomized control trial was conducted at a public primary care clinic in Singapore. Thirty children, aged 4–10 years were randomized to an intervention group (*n* = 15) using VR and a control group (*n* = 15) without VR during immunization. Feasibility was assessed by the response rate to the use of VR. The Faces Pain Scale-Revised (FPS-R) and the Children’s Fear Scale (CFS) were used to determine their pain and anxiety, respectively. The anxiety level of their accompanying parents and attending nurses were evaluated using Visual Analog Scale (VAS) prior and post-immunization of these children. The FPS-R and CFS scores, and anxiety assessment for parents and nurses were assessed using Mann-Whitney *U* test. Wilcoxon signed rank test was used to assess the difference in the nurses’ experience of using the VR application.

**Results:**

One child refused to use the VR equipment, constituting a rejection rate of 6.7% (1/15) but no adverse event occurred in the intervention arm. The overall response rate of 88% (30/34) when the parents were approached to participate in the study, indicating feasibility of using VR in childhood immunization. In the intervention group compared to the control group, the change in scores for CFS (median −1, IQR −2 to 0; *P* = 0.04), parental VAS (median −4, IQR −5 to −1; *P* = 0.04) were significantly decreased. After immunization, nurses scored favorably for VR, in terms of simplicity (median 9.5, IQR 5.72 to 10; *P* = 0.01), acceptability (median 10, IQR 5 to 10; *P* = 0.005) and willingness to use VR in the future (median 10, IQR 5 to 10; *P* = 0.02).

**Conclusion:**

Immersive VR is feasible, safe and effective in alleviating anxiety among the children and parents. Nurses viewed the application of VR in childhood immunization favorably.

**Clinical Trial Registration:**

[https://clinicaltrials.gov/ct2/show/NCT04748367], identifier [NCT04748367].

## Introduction

Pediatric medical procedures often result in significant level of fear and anxiety among the children. Immunization is a short procedure commonly performed during childhood to protect the child against common infectious disease by boosting their immunity. However, the immunization procedure using syringes and needles often results in pain, triggering phobia and distress in children (1). The resultant unpleasant experience by the children also leads to anxiety among their accompanying parents and is associated with subsequent vaccine hesitancy (2).

In 2019, the World Health Organization (WHO) announced vaccine hesitancy as one of the top 10 threats to global health (3). Pediatricians from the American Academy of Pediatrics found that 75% of parents sought to delay immunization due to concerns of their child’s discomfort (4). Approximately, 45% of the children aged 4–6 years showed serious distress during immunization (5).

The WHO has also raised the alarm on the influenza pandemic (3), which results in significant morbidity and mortality. Children with influenza often lead to influenza outbreaks in adults. Annual mass vaccinations are needed to elevate the herd immunity to the seasonal flu and tremendously reduce its effect on the morbidity and mortality of the population. Children need yearly influenza vaccination to protect them from the rapidly evolving strains, which can spread readily in childcare facilities and schools. However, adherence to the annual immunization in children has always been challenging due to their pain experience and anxiety during immunization (6). In a cross-sectional study on childhood seasonal immunization in Singapore concluded that parental knowledge on influenza illness and willingness to vaccinate were high, but influenza vaccine uptake in children remained low in the study population (7). Distress and fear in children and their parents or caregivers frequently result in default to their recommended immunization schedule. During the recent COVID-19 pandemic, addressing children’s and parents’ anxiety during immunization becomes critical for the successful roll out of COVID-19 vaccination among the children. COVID-19 vaccine has to be administered in two separate doses to the children. Challenges are expected in completing the COVID-19 vaccination if the children have unpleasant experience when they receive their first dose. Vaccine hesitancy by both the children and their parents may emerge and requires our serious attention (5, 8).

The need to search for an acceptable solution to overcome this immunization barrier becomes imperative. Enhancing the pleasant experience of children undertaking the vaccination is postulated to ease their fear and anxiety. The favorable outcome may facilitate the annual uptake of influenza vaccination and those related to other life-threatening childhood infective diseases such as measles, mumps, rubella, and varicella.

Distraction is a common non-pharmacologic technique used to attenuate pain and anxiety during painful medical procedures in pediatric patients (9). Both passive distraction (e.g., watching television, listening to music) and active distraction (e.g., interactive toys and electronic games) have been shown to reduce pain and anxiety in variable degrees (9). Nevertheless, in these interventions, the child remains cognizant of the surrounding and the presence of the healthcare professional with the syringe, which can trigger their anxiety.

Virtual reality (VR) is an interactive computer-based system that immerses the user in a three dimensional simulated environment. The VR technology in healthcare offers a potential solution to reduce distress and fear in children undertaking immunization. It completely immerses the children in a virtual environment, involving their visual-auditory and other senses (10). The child is required to don a head-mounted VR device which completely obliterates their vision to the surrounding environment. By diverting their attention to an attractive virtual surrounding, the pain signals can potentially be reduced during the vaccination procedure. Gold et al., postulated that VR-related analgesia results from the inter-cortical modulation of the pain signaling pathways via the attention, memory, emotion and other senses (i.e., visual, auditory, and touch) to mitigate pain (11).

Virtual reality has been used to enact virtual analgesia, such as in adjunctive pain control during repetitive dressing procedure in patients with burn wound (12). For the past decade, VR had been deployed in clinical trials to reduce perioperative anxiety and pain successfully in pediatric patients during procedures, ranging from dental and oncological care to intravenous needle access (13–19). VR usage in pediatric procedural pain and anxiety is rapidly evolving (17, 18). The two common VR approaches are distraction and procedural preparation (18). Immersive communication using VR has shown to address vaccine hesitancy in adults by increasing their understanding of immunization (20). This impact will have possible implications on addressing vaccine hesitancy among parents and their children (20). A recent systematic review and meta-analysis concluded that VR mitigates procedural pain and anxiety in pediatrics, but it also reported high risk of bias across studies and significant publication bias (18). Currently, robust evidence on the effectiveness of VR to alleviate pain and anxiety during childhood immunization is limited (21–23). While Rudnick Chad et al., had shown pain and fear alleviation in children wearing a VR headset, it was a feasibility study with a single arm (21). Another cross-sectional (non-randomized) study had shown reduction of fear and pain among children aged 4–6 years old when VR was used during immunization (23). Little information is available on the design and content of the VR which could confer the benefit of the virtual analgesia. A recent systematic review by Smith et al., alludes to pain alleviation using VR but suggests the need for individualized pilot testing in specific clinical use (17). In addition, a qualitative study that highlighted the importance of personalizing the VR designs by multiple stakeholders, including input from the children (24).

The literature on the nurse’s anxiety during childhood immunization is sparse. A study conducted by Jensen et al., showed that using VR simulation in nursing training could reduce nursing student’s anxiety in performing intravenous cannulation (25). Offering immersive VR as an option in childhood immunization program requires the support of the professionals such as the nurses at the healthcare facility. Nehring et al., had described the use of VR simulation in nursing education in the past 3 decades. It is well accepted by the nursing students due to the interactive feature and the ability to provide feedback (26). However, the perspectives of practicing nurses deploying VR in medical procedures are unknown.

In Singapore, the important childhood vaccinations in the National Children Immunization Schedule (NCIS) are largely implemented in both the public primary care clinics (polyclinics) and the private General Practitioner (GP) clinics. In the polyclinics, such vaccinations are routinely administered by primary care nurses using the conventional method. VR is novel in its application in local community pediatric services in primary care, even though it may be relatively well established in other countries. Parental acceptance of VR during vaccination of their older children has yet to be assessed. Likewise, the willingness of the primary care nurses to use the newer technology when they vaccinate children needs evaluation.

The VR usage in childhood immunization was postulated to be feasible in terms of acceptance by the parents and their children and that the nurses would accept its deployment during the procedure. VR was also hypothesized to alleviate pain and anxiety in children during their immunization and would reduce their parental anxiety compared to usual care.

This study primarily aimed to determine the feasibility of using immersive VR during immunization in children. The secondary outcomes were the difference in children’s pain score between intervention and control group, the change in the anxiety levels of the children, their parents and their attending nurses before and after their immunization based on the scores of the validated scales. In addition, this study aimed to assess the acceptability and willingness of the nurses and their perceived ease of VR usage during the immunization. This study also aimed to assess the willingness of the children and their parents in both groups to undertake future immunization. This feasibility study will provide the relevant information to design a culturally adapted and adequately powered randomized controlled trial.

## Materials and Methods

Due to the novelty of VR application in pediatric procedures in Singapore, this study was conceptualized as a proof of concept feasibility trial to examine the VR analgesia in childhood immunization in primary care. This single-center, open label, randomized controlled trial was also designed to evaluate the preliminary clinical outcomes of the children and their parents.

### Study Setting

This study was conducted at a regional public primary care clinic (polyclinic) in Sengkang in north-east Singapore, which serves about 240,000 residents. Children under the age of 9 years account for 14.5% of the estate residents, comprising the second highest pediatric population in Singapore (27).

### Study Population

The study population included multi-ethnic Asian children aged 4–10 years, their parents and the registered nurses who administered their immunization at the polyclinic. Children aged 4–10 years tend to be apprehensive during immunization due to their cognitive and emotional development, and are postulated to benefit from the VR intervention. In addition, the head circumference of these children to fit the VR headset, which weighs 570 grams, is a major consideration.

Literature suggests that young children, aged 4 to 10 years, tolerate fully-immersive 3D virtual reality game play (two sequential play sessions and each lasting 30 min) without significant effects on their visual-motor functions, post-VR postural instability or maladaptation of the vestibule-ocular reflex (28). The prevalence of discomfort and side effects are reported to be lower than the adverse effects for adults (28).

The children included those of both gender and of any ethnicity, who were accompanied by their parents (mother, father or both) or legal guardians. The registered nurses were those who were assigned routinely to carry out immunization at the respective service room based on the polyclinic nursing duty roster.

Children with pre-existing epilepsy/seizure or migraine and those with disability which rendered them incapable of providing assent were excluded. Children that were not accompanied by legal guardian to provide consent were also not recruited.

### Sample Size Calculation

Julious SA recommends a sample size of 12 per group as a rule of thumb for a pilot study (29). To allow for dropouts during the immunization, 30 children would be recruited for this pilot study, with 15 in the intervention group (VR) and 15 in the control group.

### Randomization

Randomization was performed *a priori* using a computer generated randomization system. The randomization sequences were concealed in numbered, opaque envelopes. Blinding of the child was not possible in this study due to the nature of the intervention. After obtaining the consent and assent, the study team member retrieved the randomization envelope to reveal the group assigned to the child.

### Evaluation of Feasibility

Feasibility was evaluated based on the recruitment response rate to the study, regarded as a surrogate indicator of their acceptance to the use of the new technology. This was computed based on the number of parents who consented to the study participation among those who were potentially eligible when they were approached by the clinical research coordinators (CRC). The incidences of adverse effects when VR was used during the immunization and rejection rate by the child to its use are additional indicators for feasibility.

### Instruments to Assess Clinical Outcome

Self-reported measurement tools have been used to assess pain in children. The Faces Pain Scale-Revised (FPS-R), a validated and well-established psychometric tool, was selected for this study based on the recommendation by earlier studies and the International Association for the Study of Pain (IASP) (30–33). The FPS-R scale has strong positive correlation with the visual analog scale (VAS) in children aged 4 to 16 years (30, 32).

To date, several validated, self-reporting tools are available to assess the children’s’ anxiety (34, 35). The McMurtry Children’s Fear Scale (CFS), adapted from the Faces Anxiety Scale originally used in adult, was chosen for this study. CFS has shown satisfactory construct validity, test-retest reliability and inter-rater reliability in a previous study which assessed anxiety during a needle procedure in children (35). Both the FPS-R and CFS are validated scales to be used in children from the age of 4 years and older.

The CRC assisted in the questionnaire administration. The CRC explained the FPS-R and CFS scales to the children using a standardized script. They ensured that the child understood the instructions before the child rated his or her pain and anxiety using the validated scales, respectively. It took approximately 2 min for each child to complete both scales. On average, the assessment of baseline anxiety was carried out within 2 min before immunization. After immunization, the assessment of pain and anxiety for children were done almost immediately (within 2 min) in order to capture their actual experienced pain and anxiety during immunization.

Visual Analog Scale (VAS) was used to assess anxiety of their parents and attending nurses before and after the immunization. Likert scales (range of 1–10) were used to assess the acceptability, willingness and perceived ease of use of the VR technology, respectively by the nurses in the intervention group. The same Likert scale (range of 1–10) was also being used to assess the children’s and their parents’ willingness to return for future immunization.

### Virtual Reality Software

Evidence suggests that if the VR environment is individually tailored to the user’s age, gender, ethnicity, the VR analgesic effect will be enhanced (11, 12). The VR software named **SILVER** (**S**oothing **I**mmunization **L**everaging on **V**irtual Reality **E**xpe**r**ience) was co-created by the two investigators with software engineers and the virtual artist team from the AI Innovation Labs in Singapore.

A *de novo* VR mascot and setting were created specifically for the SILVER software in the design-thinking phase of the trial. Feedbacks were initially collated from 20 children aged between 4 and 10 years, to select one of three new mascots when they attended the polyclinic (study site) prior to the study. The mascot called Burp was eventually chosen as it was well-liked by these children, regardless of their gender. Burp is a little yellow creature with long floppy ears wearing a blue witch hat and holding a magic wand.

**FIGURE 1:**
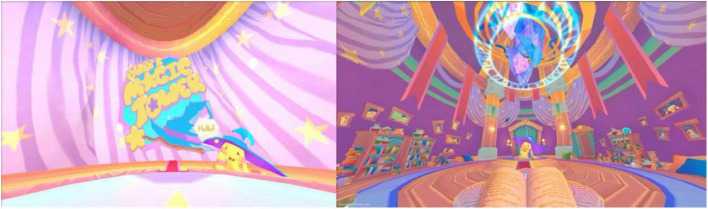
The Burp’s Magical Tower depicted the SILVER software.

The SILVER software enacts a story centered on the “Burp’s Magic Tower” to portray a cosy room filled with shelves of books and other magical items. It differed from the clinical setting in an immunization room. Once the child wears the VR headset, the child can see a spell book with a giant blue crystal tower floating above. Burp will inform the child to assist him to power up his crystal tower. The duration of the storyline is about 2 min. About a minute into the story, Burp uses the magic wand to tap on the child’s left shoulder which coincides with the point of injection. The rune on the child’s left shoulder is activated to enable magical power to flow from it to the crystal tower. Figure 1 shows snippets of the software.

### Virtual Reality Equipment

The VR equipment comprises an Oculus Quest headset (Facebook Technologies), which measures 8.7 × 7.6 × 4.1 inches and weighs 0.57 kg. The field of angle is 100°, with adjustable inter-pupil-distance (IPD) ranging from 58 to 72 mm. The VR headset has an adjustable strap which can fit various head sizes. In the intervention group, the nurse was responsible to turn on the VR device, mounted it on the child’s head and administered the vaccine while paying attention to the animation on the tablet. A CRC was in the vicinity to provide technical support if required. The nurses and CRCs were trained by the Principal Investigator (PI) and a software engineer from the industrial partner (AI Innovation Labs). The latter inducted the study team members to manage the VR equipment and to solve emerging technical issues.

### Recruitment Procedure

The CRC screened the list of children scheduled for their influenza or other childhood immunizations for recruitment eligibility daily at the study site. When the child turned up for their immunization appointment, the CRC provided the parent or legal guardian with the study information and clarified their doubts before seeking their consent. They also spoke to the child on the potential use of the VR gadget to seek their assent.

Written informed consent from the parent or legal guardian and assent from children were obtained prior to their study enrollment. The study team recruited nurses assigned routinely to carry out immunization at the study site. The entire team of registered nurses fully accepted and endorsed their consent to participate in the study. The study was conducted from September to December 2020.

### Pre-intervention Procedure

The children in both groups rated their baseline anxiety score using the CFS. Their parents and respective attending nurses rated their baseline anxiety level using the Anxiety VAS before the immunization. The immunization room was cleared of any audio-visual equipment to minimize any distraction to the child. The allocations (intervention vs. control group) were only revealed after the consent-taking and the participants had scored their pain and anxiety levels using the scales.

### Intervention Group

After revealing the allocation, the children in the intervention group were briefed on the cartoon animation. After the children entered the immunization room, the children donned the VR headset and viewed the VR animation. The attending nurse concurrently viewed the animation via a tablet. At a specific juncture, the nurse administered the vaccine to the child. The duration of the animation was about 2 min. At the end of each immunization, the VR headset would be cleaned and sanitized according to the standard infection control measures at the study site.

### Control Group

The children in the control group underwent immunization according to the institution standard operating protocol. The nurse explained the immunization procedure to parents and children before the administration of the vaccine. The children in the control group were allowed to use other distractions during immunization such as electronic devices, toys, books, and listening to music. Parents or additional nurses were instructed to hold the child, depending on the children’s anxiety level to ensure safety during the vaccine administration.

### Reimbursement

Only the children (accompanied by their parents) were reimbursed with SGD20.00 (estimated USD15.00) for spending their time to participate in this study.

### Statistical Analysis

All analyses were performed using IBM SPSS version 25.0. Both Intention to treat (ITT) and Per Protocol (PP) analysis were performed. In view of the small number of children in both groups, Mann-Whitney U test was used to assess the difference in the change in the median anxiety and pain score in children, change in the median scores of the parental and nurse’s anxiety scores, respectively. Chi-square test and Fisher’s exact test were used to assess the difference in the demographics of the two groups. Wilcoxon signed rank test was used to assess the difference in the nurses’ experience of using the VR application. A *P* value of less than 0.05 is considered statistically significant.

### Ethical Approval and Funding

Ethics approval was granted by the SingHealth Centralized Institutional Review Board (CIRB reference number: 2019/2857). The study was conducted in compliance with the ICH Guideline for Good Clinical Practice (GCP) and Human Biomedical Research Act (HBRA). This pilot study was funded by the Academic Medicine Philanthropic Fund and seed funding from SingHealth DUKE-NUS Family Medicine Academic Clinical Program (FM ACP). AI Innovation Labs developed the SILVER software as an in-kind contribution in this project.

## Results

**FIGURE 2:**
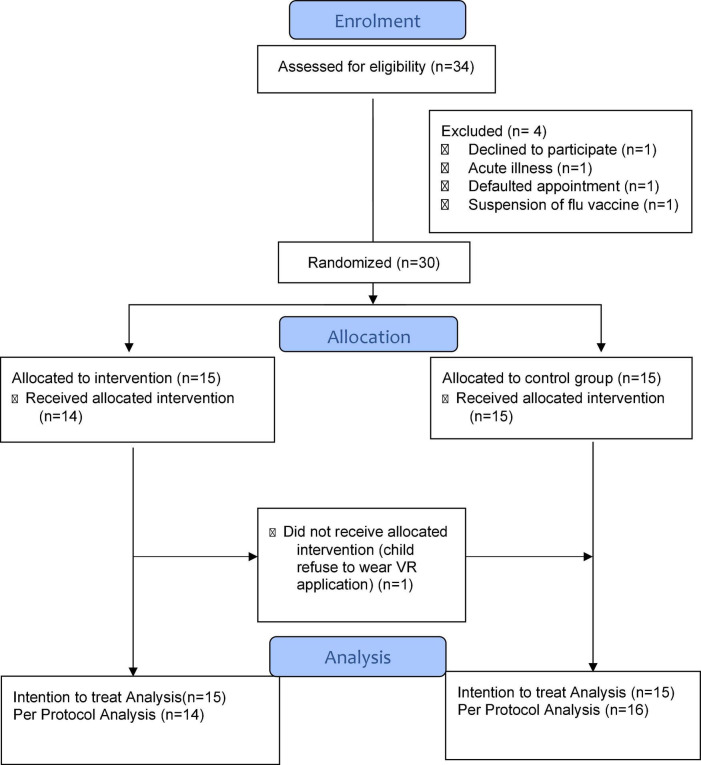
CONSORT (Consolidated Standards of Reporting Trial) flow diagram of participants.

Between September to December 2020, 34 eligible children were screened before immunization appointment and 30 of them were recruited. The response rate of children and parents who consented to the study was 88% (30/34). Only one participant rejected to participate in the study (Figure 2). The remaining 3 participants were excluded due to acute illness, default on day of immunization and failure to proceed with vaccination during a period of temporary official suspension of influenza vaccination amidst adverse reports in South Korea. All immunization procedures in the study were video-recorded as additional measure to monitor for any emerging adverse reactions. No adverse event occurred amongst all the 30 children, including those in the VR intervention group. One child was originally randomized to the intervention group but refused to don the VR equipment (Figure 2). Therefore, both intention-to-treat (ITT) and per protocol (PP) analyses were performed to determine any significant differences in the clinical outcomes.

Table 1 depicts the similar baseline demographic characteristics of the children and their parents in both intervention and control groups, except for gender. More male children (*n* = 19, 63.3%) were recruited in the study and 13 (86.7%) of them were present in the control group based on the original allocation.

**TABLE 1:** Baseline characteristics of children and parents (*n* = 30).

		Per protocol			Intention to treat		
	Total	Intervention	Control	*P* value	Intervention	Control	*P* value
**Total children, *n* (%)**	30 (100.0)	14 (46.7)	16 (53.3)		15 (50.0)	15 (50.0)	
**Age, median (IQR)**	6 (5–8)	6 (5–8)	7 (4.25–7.75)	0.69	6 (5–8)	7 (5–8)	0.90
**Gender**				0.007			0.008
Male	19 (63.3)	5 (35.7)	14 (87.5)		6 (40)	13 (86.7)	
Female	11 (36.7)	9 (64.3)	2 (12.5)		9 (60)	2 (13.3)	
**Ethnic group**				0.44			0.70
Chinese	20 (66.7)	8 (57.1)	12 (75)		9 (60)	11 (73.3)	
Malay	10 (33.3)	6 (42.9)	4 (25)		6 (40)	4 (26.7)	
**Vaccine name**				> 0.99			0.71
Chickenpox	17 (56.7)	8 (57.1)	9 (56.3)		9 (60)	8 (53.3)	
Influenza	13 (43.3)	6 (42.9)	7 (43.7)		6 (40)	7 (46.7)	
**Route of administration**				> 0.99			0.71
Subcutaneous	17 (56.7)	8 (57.1)	9 (56.3)		9 (60)	8 (53.3)	
Intramuscular	13 (43.3)	6 (42.9)	7 (43.8)		6 (40)	7 (46.7)	
**Parent’s age, median (IQR)**	37 (34.5–40)	35.5 (30.5–37.75)	37.5 (36–40)	0.06	36 (31–39)	37 (36–40)	0.10
**Gender of parent**				> 0.99			>0.99
Male	5 (16.7)	2 (14.3)	3 (18.8)		3 (20)	2 (13.3)	
Female	25 (83.3)	12 (85.7)	13 (81.3)		12 (80)	13 (86.7)	
**Ethnic group**				0.21			0.28
Chinese	20 (66.7)	8 (57.1)	12 (75)		9 (60)	11 (73.3)	
Malay	7 (23.3)	3 (21.4)	4 (25)		3 (20)	4 (26.7)	
Indian	3 (10)	3 (21.4)	0 (0)		3 (20)	0 (0)	
**Highest educational level attained by parents**				0.48			0.76
Secondary	3 (10)	2 (14.3)	1 (6.3)		2 (13.3)	1 (6.7)	
A-level/diploma (ITE/polytechnic/private school)	17 (56.7)	9 (64.3)	8 (50)		9 (60)	8 (53.3)	
University/post-tertiary	10 (33.3)	3 (21.4)	7 (43.8)		4 (26.7)	6 (40)	

No difference on the children’s pain score was reported between the two groups based on the ITT analysis (*P* = 0.13) but statistical significance (*P* = 0.04) can be found using the PP analysis (Table 2). The change in children’s fear score was significantly reduced in the intervention group compared to the control group in both the PP (*P* = 0.02) and ITT analysis (*P* = 0.04). Similarly, the change in parental anxiety score was significantly decreased in the intervention versus the control group regardless of the type of analysis. The change in nurse’s anxiety scores showed no significant difference between the two groups (Table 2).

**TABLE 2:** The children’s pain score, the change in the children’s fear scores, nurse’s and parental anxiety scores in the intervention and control groups.

	Per protocol			Intention to treat		
	Intervention	Control	*P* value	Intervention	Control	*P* value
Child’s Pain Score (FPS-R), median (IQR)	0.5 (0–2)	2 (0–10)	0.04	1 (0–2)	2 (0–10)	0.13
Change in Child’s Fear Scale (CFS), median (IQR)	−1 (−2.25–0)	0 (−0.75–1.5)	0.02	−1 (−2–0)	0 (−1–2)	0.04
Change in Parent’s Anxiety Score, median (IQR)	−4 (−5 to −2.5)	0 (−3.75–2)	0.009	−4 (−5 to −1)	0 (−4–2)	0.04
Change in Nurse’s Anxiety Score, median (IQR)	−1 (−2.5–0)	−1 (−3–0)	0.81	−1 (−2–0)	−1 (−3–0)	0.51

After using the VR procedure during the immunization, nurses in the intervention group perceived that the VR application was simple (median 9.5, IQR 5.75 to 10; *P* = 0.01), acceptable (median 10, IQR 5 to 10; *P* = 0.005) and were willing to use VR (median 10, IQR 5 to 10; *P* = 0.02) in the next immunization (Table 3).

**TABLE 3:** Nurses’ perspectives of using VR in childhood immunization (intervention group).

	Before	After	*P* value
Simplicity of VR application, median (IQR)	5 (4–10)	9.5 (5.75–10)	0.01
Acceptability of VR application, median (IQR)	9 (4.75–10)	10 (5–10)	0.005
Willingness to use the VR application in the next immunization, median (IQR)	9.5 (4–10)	10 (5–10)	0.02

More children were willing to return for future immunization after the procedure in the intervention group (*n* = 11, 73.3%) compared to control group (*n* = 6, 40%) but the difference did not attain statistical significance in both the ITT or PP analyses (Table 4). No difference was noted in the parental willingness to bring their children for immunization (Table 4).

**TABLE 4:** Children’s and parent’s willingness to proceed with future immunization.

			Per protocol			Intention to treat		
	Total	Intervention	Control	*P* value	Intervention		Control	*P* value
**Children’s willingness to come for immunization in the future**								
Yes	17 (56.7)	10 (71.4)	7 (43.8)	0.16	11 (73.3)		6 (40)	0.14
No	13 (43.3)	4 (28.6)	9 (56.3)		4 (26.7)		9 (60)	
**Parent’s willingness to come for immunization in the future**								
0 to 10, Median (IQR)	10 (9–10)	10 (9.75–10)	10 (9–10)	0.45	10 (10–10)		10 (9–10)	0.33

Comparison of post pain score and change in anxiety scores between the children aged 4–7 years old and the older children aged 8–11 years old were shown in Table 5. No difference was found in their pain score and anxiety score between the two groups (Table 5).

**TABLE 5:** Association of age with scores.

	4–7 years old	8–10 Years old	*P*-value
Post Child’s Pain score	1.5 (0–7)	2 (0.5–2)	0.587
Change in Child’s anxiety score	0 (−1.25–0)	−1 (−2–0)	0.476

After adjusting for baseline scores, gender and age in the per protocol (PP) analysis, both the pain score and fear score are significantly lower in the intervention group compared with the control group. However, only the fear score is significantly reduced in the intention-to-treat (ITT) analysis (Table 6).

**TABLE 6:** Association of post pain and fear score with intervention using linear regression.

Variable	Beta (95% CI)	*P*-value	Assessment of direction
**ITT**			
*Post Child’s Pain Score (FPS-R)	−1.787 (−4.395–0.821)	0.179	Intervention < Control
*Post Children’s Fear Scale (CFS)	−1.331 (−2.385 to −0.277)	0.013	Intervention < Control
**PP**			
*Post Child’s Pain Score (FPS-R)	−3.29 (−5.846 to −0.733)	0.012	Intervention < Control
*Post Children’s Fear Scale (CFS)	−1.253 (−2.366 to −0.141)	0.027	Intervention < Control

*Adjusted for baseline scores, gender, and age.

## Discussion

This study shows that immersive VR application in childhood immunization is feasible in primary care in Singapore. The technology seems to be acceptable by the end users, namely the healthcare professionals, children and their parents. The response rate was high in this study despite the tool being relatively novel in clinical practice in Singapore. Adult residents in the local urban community are cognizant of VR via various media such as television programs and movies, and are probably receptive to try it out on their children.

Previous literatures suggested the potential benefits of VR in reducing procedural pain and anxiety in children (18). However the effect of VR in pediatric procedures were mainly observed in dental procedures and other inpatient needle-related procedures such as venipuncture, IV cannulation and port access (15, 18, 19). The evidence for VR usage in immunization, is limited (21–23). This present study has shown the feasibility of using VR in childhood immunization, with potential effectiveness in alleviating the pain and anxiety in immunization among children. A future RCT with customized VR design incorporating multiple stakeholders perspective is required to examine the effectiveness of VR in reducing pain and anxiety during immunization among children (24). In this study, children in the target age group had selected the mascot in the VR software. As the major users, these children will continue to contribute to the design and development of future VR applications.

Adverse events of using VR in children were rare based on current literature (15, 17, 28). None of the children experienced any adverse effect from using the VR in this study. Only one young child (4-year-old) was fearful and refused to wear the headset, which might be due to lack of previous exposure to VR equipment. Overall, the refusal rate seems to be low in this pilot study. Explanation of the procedure to the children, as an essential step when assent was obtained, could also allay their concern and enhance their uptake of the VR prior to the immunization.

The children’s pain score reached statistical significance while using PP analysis (Table 2). Pain assessment in children remains a challenge and self-reported pain assessment via validated FPS-R scale was used in this study. Despite the immersive VR, pain can still be experienced by children during the immunization. The feasibility study by Rudnick Chad et al., used the Wong-Baker Pain Scale, which is another self-reported instrument (21). They had reported pain alleviation using VR in their single arm study (21).

The pain perception by children evolves through the stages of their cognitive development based on the Piaget Developmental Model. The children’s pain perspective progressed from pre-operational stage (2 to 7 years) to concrete operational stage (7–11 years), before transiting to more complex notions of pain perception (36–38). Nonetheless, the subgroup analysis on two groups of children who were 4–7 years old and compared with those older children aged 8–11 years old revealed no difference in their pain score and anxiety score. Nordgard R et al., also found no relationship between the participant age and the effects of VR on pain score or anxiety score ‘(18).

The clinical outcomes in this study revealed significant decline in anxiety level among children and their parents. Such positive outcome seemed to reduce their apprehension toward future immunization. Heden et al., found that fear levels were higher than pain levels during needle insertion in subcutaneous implanted port amongst pediatric oncological patients with application of topical anesthesia. Fear-reducing intervention seems to be an important measure in needle procedures (39). Hence, the use of immersive VR during immunization alleviates anxiety among the children, created pleasant experience and may potentially raise the uptake of repeated seasonal vaccines, such as influenza immunization.

The study population targeted children who were scheduled for their immunization, which suggested that the parents were receptive and recognized the merits and values of such preventive measure. The majority of the parents are also tertiary educated (Table 1). The results showed the parental willingness to continue with future immunization for their children (Table 4). Despite the self-selected study population in terms of the parental educational background and preferences, any potential bias in assessing the VR application in childhood immunization was mitigated by the randomization of these children in this study. More children in the intervention group were willing to undertake future immunization although the difference was not statistically significant.

The primary care nurses are well-trained to handle children with needle phobia. Therefore, no difference in their anxiety level was observed in both groups. Their favorable perception of the VR tool will be pivotal in scaling up its application in primary healthcare practices. VR may become an additional tool to be used in children with special needs and those with immunization phobia.

### Strength

The strength of this pilot randomized controlled trial is exemplified by its demonstration of the feasibility of using the VR when children aged 4 years and above are immunized. This study also showed promising outcomes in reducing the anxiety of the children and their parents. The VR content and mascot Burp were created with design thinking, with direct feedbacks from the children themselves. Concurrently the study also sought the perspectives from the primary care nurses, who would be the key providers of VR-based childhood immunization in the future. Their favorable views have enhanced the translational potential and scalability of this innovation when it is introduced in general or primary care practices.

### Limitations

The VR application in immunization inevitably adds initial cost to the procedure to cover the procurement of the VR equipment and development of the VR software. Cost-effectiveness of applying this healthcare technology in clinical practice has yet to be examined. Nonetheless, the economic savings to society and disability-adjusted life years saved in children when infectious disease outbreaks or even epidemics are curbed are expected to be significant.

The children and their parents were not blinded to the VR intervention. Ideally the children in the control group could wear the headset without experiencing any immersive VR. However, the study team aimed to compare the effect and acceptability of the VR with routine immunization procedure, with or without other means of distraction commonly used by their parents.

More boys were recruited in the control group as the randomization was not gender-stratified, which could limit the generalizability of the results. Whether there is gender difference in anxiety and pain perception is unclear but may be addressed by gender stratification in future randomized controlled trial. The findings in this study will be utilized to calculate the sample size to ensure adequate power for such a trial.

### Clinical Implications and Considerations

The application of VR in the local pediatric population is relatively novel in primary healthcare setting. In this study, the polyclinic nurses underwent training by the study team, which was essential to enhance their competency and confidence in using the VR equipment before administering the vaccine. Technical support by the VR software development team is also vital to ensure effective implementation of the innovation.

A library of VR software with different content and storylines may be needed to cater to children who require repeated immunization with seasonal vaccines. The intent is to sustain their interests in using VR and to create favorable experience for them and their parents during subsequent immunizations. Such resource material will allow the assessment of virtual analgesia effectiveness in repeated childhood immunization.

In the current COVID-19 pandemic, Singapore has rolled out COVID-19 vaccination for children aged 5–11 years old since January 2022. The use of VR has potential impact in mitigating the children’s pain experience and anxiety when they receive their Covid-19 vaccination and reduce vaccine hesitancy among their parents.

## Conclusion

Immersive VR intervention during immunization was feasible, safe and effective in alleviating anxiety among the children and parents. Children reported higher pain alleviation and were more willing to undertake subsequent immunization using VR, although the differences did not attain statistical significance. The attending nurses accepted and were willing to use this new technology, perceiving it to be simple to be deployed during childhood immunization.

## Data Availability Statement

The original contributions presented in the study are included in the article/supplementary material, further inquiries can be directed to the corresponding author.

## Ethics Statement

The studies involving human participants were reviewed and approved by SingHealth Centralized Institutional Review Board (CIRB). Written informed consent to participate in this study was provided by the participants’ legal guardian/next of kin.

## Author Contributions

ZC and NT were responsible for the study design, writing the protocol, contributing ideas in VR software development, interpretation of the result, and revising and editing the manuscript. JT and CG were responsible in contributing ideas and developed the VR software that suited the clinical needs in this study. EK was responsible for the study design, conducted the statistical analysis, interpreted the results, and wrote and reviewed the manuscript. GK and RF facilitated the patient recruitment and responsible for revising and editing the final manuscript. All authors contributed to and approved the final manuscript.

## Conflict of Interest

The authors declare that the research was conducted in the absence of any commercial or financial relationships that could be construed as a potential conflict of interest.

## Publisher’s Note

All claims expressed in this article are solely those of the authors and do not necessarily represent those of their affiliated organizations, or those of the publisher, the editors and the reviewers. Any product that may be evaluated in this article, or claim that may be made by its manufacturer, is not guaranteed or endorsed by the publisher.

## Abbreviations

CFS: Children’s Fear Scale
CRC: clinical research coordinators
FPS-R: Faces Pain Scale-Revised
ITT: intention to treat
PP: per protocol
SILVER: soothing immunization leveraging on virtual reality experience
VAS: Visual Analog Scale
VR: virtual reality
WHO: World Health Organization.
